# Development of a brief, generic, modular resource-use measure (ModRUM): cognitive interviews with patients

**DOI:** 10.1186/s12913-021-06364-w

**Published:** 2021-04-21

**Authors:** Kirsty Garfield, Samantha Husbands, Joanna C. Thorn, Sian Noble, Will Hollingworth

**Affiliations:** 1 Health Economics at Bristol, Population Health Sciences, Bristol Medical School, University of Bristol, 1-5 Whiteladies Road, Bristol, BS8 1NU UK; 2 MRC ConDuCT-II Hub for Trials Methodology Research, Population Health Sciences, Bristol Medical School, University of Bristol, Bristol, BS8 1NU UK

**Keywords:** Resource-use measurement, Self-report, Questionnaire development, Cognitive interview, Think-aloud interview, Content validity

## Abstract

**Background:**

Self-report resource-use measures (RUMs) are often used to collect healthcare use data from participants in healthcare studies. However, RUMs are typically adapted from existing measures on a study-by-study basis, resulting in a lack of standardisation which limits comparability across studies. Psychometric testing of RUMs is rarely conducted. This paper reports on cognitive interviews with patients to test the content validity and acceptability of a new RUM (ModRUM). ModRUM is a brief, generic RUM with a core module on healthcare use and questions/modules to increase depth and breadth.

**Methods:**

A purposeful sampling strategy with maximum variation was used to recruit patients from primary care to participate in “think-aloud” interviews with retrospective probing. Participants verbalised their thought processes as they completed ModRUM, which allowed errors (issues with completion) to be identified. The interviewer asked follow-up and probing questions to investigate errors, clarity and acceptability.

Interviews were audio-recorded and transcribed verbatim. Research team members independently scored transcripts to identify errors in comprehension, recall, judgement and response. Members met to agree on final scores. Interview transcripts were analysed qualitatively using techniques of constant comparison, to identify common themes and ideas for improvement. Data collection and analysis were performed concurrently and in rounds.

**Results:**

Twenty participants were interviewed between December 2019 and March 2020. Interviews were conducted in three rounds, with revisions made iteratively and in response to interview findings. Seven participants completed the core module and 13 completed the core module plus depth questions. Of 71 issues, 28 were in comprehension, 14 in retrieval, 10 in judgement, 18 in response and 1 uncategorised. Most issues (21 issues by 2 participants) were due to participants including family healthcare use. Other issues included using incorrect recall periods (5 issues) and overlooking questions leading to missing responses (9 issues). Common participant suggestions included highlighting important details and providing additional definition or examples for some terms. The length, content and layout were acceptable to most participants.

**Conclusions:**

A generic RUM is needed to increase study comparability. RUM development requires thorough testing to demonstrate and enhance validity. Cognitive interviewing has demonstrated the acceptability and content validity of ModRUM.

**Supplementary Information:**

The online version contains supplementary material available at 10.1186/s12913-021-06364-w.

## Background

Evidence from randomised controlled trials (RCTs) is often utilised by decision-making bodies when making resource allocation decisions. Within RCTs, cost-effectiveness estimates are frequently informed by participant-reported resource-use and outcomes. While validation of outcome measures is common and a wealth of literature exists on outcome measure development, psychometric testing of participant-report resource-use measures (RUMs) is not routinely carried out [1–5]. This is problematic as it limits the ability to draw valid conclusions from which to inform resource allocation decisions.

Validation of an outcome measure is a time-consuming process which involves psychometric testing [1, 2]. As new RUMs are often designed or adapted from previous RUMs on a trial-by-trial basis and within the time constraints of an RCT, validation is not commonly undertaken [5]. Where validation has been undertaken, it is often only criterion validity that is assessed by comparing RUM results to administrative data and/or medical records; however, it is usually only a subset of the RUM that can be validated [6].

A well-validated, generic RUM would avoid duplication of research and improve the quality of resource-use data captured. A standardised RUM would also enhance the comparability of results, improving the validity of comparisons of cost-effectiveness made across RCTs. The overall objective of our research is to develop and validate a new generic RUM (ModRUM – Modular Resource-Use Measure). ModRUM was developed primarily for use in the United Kingdom National Health Service (NHS) system. The core module captures data on NHS-funded healthcare delivered in hospitals (Accident and emergency visits, outpatient appointments, and inpatient and day case stays) and the community (General practitioner (GP) and other healthcare professional contacts). Depth questions capture data with more granularity (for example: clinic type, tests performed and reason for an outpatient visit) and capture data on ambulance contacts and prescribed medications. Development to date has included: item identification in an expert Delphi consensus study with 45 experienced health economists [7]; a review of existing RUMs; development of a prototype; and assessment of the face and content validity, and suitability of the prototype for costing purposes in qualitative interviews with 10 health economic experts.

Interviews can be used to both assess patients’ comprehension and evaluate comprehensiveness, to ensure questions capture the information they are intended to capture (i.e. content validity) [8]. To date, the use of interviews to explore patient comprehension in RUM development has been limited [9] and includes testing of condition-specific and proxy-completed RUMs as opposed to generic RUMs designed for completion by a wide range of patients [10, 11]. The aim of this study was to test the content validity and acceptability of ModRUM with a wide range of patients in ‘think-aloud’ interviews with retrospective verbal probing.

## Methods

### Study design

In this study patients participated in cognitive interviews, which encompassed a think-aloud exercise and retrospective verbal probing. ‘Think-aloud’ interviews, which are an established technique for assessing the content validity of outcome measures [8], involve respondents completing questionnaires while verbalising their thought processes [12]. They are advantageous as minimal input is required from the interviewer during measure completion which allows issues to be revealed while minimising interviewer-imposed bias [12]. Retrospective verbal probing can follow the ‘think-aloud’ exercise to probe on areas where patients experienced issues and on areas of interest to the researcher [12].

### Participant sampling and recruitment

Patients were recruited from primary care organisations (PCO), from a range of deprivation levels (rated one to ten using the index of multiple deprivation for 2010, where 1 is the most deprived and 10 is the least deprived), within the Bristol, North Somerset or South Gloucestershire regions of England. A purposeful sampling strategy was used to ensure ‘information-rich’ patients, who were active users of healthcare services, were recruited [13]. To reflect the wide range of patients that could complete ModRUM in an RCT context, maximum variation sampling, based on sex, age group, ethnic group, number of long-term conditions and age on leaving full time education, was used with the aim of recruiting a diverse range of patients [14]. Eligible patients attending an appointment at their PCO were introduced to the study and provided with a patient information sheet and a reply form by their clinician or a PCO receptionist. The reply form captured information on patient characteristics and number of primary and secondary healthcare contacts in the last 3 months. Patients were asked to provide contact details if they were interested in learning more about the study in a phone call with a researcher (KG). Eligible patients for the study were: (1) aged 18 or over; (2) able to understand written and verbal English; (3) a patient at one of the participating PCOs; (4) capable of giving informed consent. Patients from groups that were considered harder to reach (male, non-white ethnic groups, lower age on leaving full time education) and patients who had used secondary healthcare were initially prioritised. Subsequently, patients who were more likely to have used healthcare were prioritised. Recruitment was informed by the characteristics of previously recruited participants, to maximise variation in the characteristics of participants. Concurrent interviews and analysis allowed us to identify when ‘data saturation’ was reached, whereby additional interviews would not have identified any new issues that had not already been considered and would not have resulted in further changes to ModRUM [15].

### Data collection

**Fig. 1:**
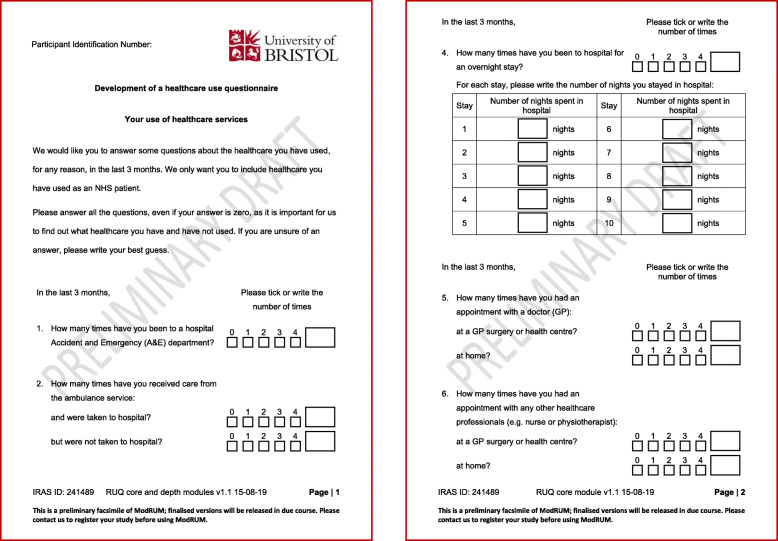
ModRUM core module: pre-cognitive interviewing

Interviews took place at a location convenient to the participants; either their home, workplace or PCO. A PhD researcher (KG), trained in qualitative research, performed the interviews. Once patients had provided written informed consent, the interview started with an established think-aloud training exercise [16], to help the participant become familiar with the ‘think-aloud’ process. The exercise involved thinking-aloud while visualising and counting windows in their home. Participants then completed the think-aloud exercise by answering (on paper) the core module (Fig. 1) or the core module plus depth questions (Additional file 1: Figure S1) of ModRUM while verbalising their thought process. Initially participants completed the core module only. Once the research team were confident that all core module specific issues had been identified, subsequent participants received the core module plus depth questions. All questions referred to healthcare use in the last 3 months. The interviewer remained silent throughout unless the participant stopped verbalising their thoughts, in which case the participant was prompted to continue speaking aloud. The ‘think-aloud’ exercise was followed by a semi-structured interview, whereby the participant was asked questions to clarify any issues that occurred and on prespecified areas of interest to the researchers including content, ease of completion and acceptability of ModRUM.

Interviews were audio-recorded, and audio-recordings were transcribed verbatim. Data were managed and/or analysed in Microsoft Excel, Stata 12 and NVivo 11.

### Data analysis

Analysis was performed concurrently with data collection, which allowed findings from earlier interviews to inform adaptations to ModRUM and the topic guide for testing in further interviews [15].

#### Data scoring

Transcriptions were analysed using a standardised classification scheme to identify response problems. The standardised classification scheme is based on the survey response model which breaks down the cognitive process of answering questions into four actions [17]. The actions include (i) comprehension of the question in the intended way; (ii) retrieval of the appropriate information from memory; (iii) judgement of how the information should be used to answer the question; and (iv) formatting the information into a valid response. For each participant, three raters (KG, SH, JCT) were provided with the transcript and participant-completed ModRUM. Raters independently scored responses by reporting for each question whether errors in comprehension, retrieval, judgement, or response occurred. Raters also noted when participants appeared to struggle with a question but were able to reach the correct answer (e.g. re-reading the question) [18]. Error classifications were made in a hierarchical order; for example, if a comprehension error was scored then no further errors or a struggle were identified. Inter-rater agreement was assessed using Gwet’s agreement coefficient with user-defined weights^1^ [19, 20]. Strength of agreement is usually considered to be substantial/excellent for agreement coefficient scores above 0.6 and almost perfect for scores above 0.8 [21]. Raters met to compare scores. Where scoring differences arose, raters discussed the scoring until they reached consensus on a final score.

#### Qualitative data coding

Transcriptions were also analysed qualitatively by KG. Techniques of constant comparison were utilised to compare participants’ comments on aspects of the RUM design, to develop key patterns and themes from participant responses and to enhance understanding of key issues experienced during RUM completion [22, 23]. Analysis involved line-by-line coding of transcripts, with data organised into themes and assigned a representative code. A coding structure was developed and applied to all interview transcripts, with codes continually updated for new data.

## Results

Five PCOs, with a range of deprivation levels (from the 2nd to 10th deprivation decile), were recruited to participate in the study. Of 58 patients who expressed an interest in taking part, 39 patients, who responded to a telephone call within the timeframe of the study, were invited to participate, and 29 patients agreed to take part. Nine interviews were subsequently cancelled by the patient due to illness or the interview time no longer being convenient, leaving 20 patients who participated in the study.

Interviews took place at participants’ homes (*n* = 14), offices (*n* = 3) or PCOs (*n* = 3). Interviews were conducted between December 2019 and March 2020 and lasted on average 26 min (range: 10–54 min). There were three rounds of interviews, with revisions to ModRUM made between and during rounds. Once the research team were satisfied with feedback and modifications made to the core module, subsequent participants (participants 9 to 20) completed the core module plus depth questions. Participant characteristics are presented in Additional file 1: Table S1.

A summary of participant healthcare use, as captured in ModRUM, is provided in Additional file 1: Table S2. On average, participants reported 2.8 (SD 1.5) appointments with the GP at the GP practice over the last 3 months. All participants who completed the prescribed medication question reported that they had picked up or received prescribed medications. Outpatient appointments were the next most frequently reported healthcare with 14 (70%) participants reporting appointments and an average of 1.4 appointments (SD 1.5). Few (< 3) or no participants reported using the following healthcare: hospital day case and inpatient stays; GP, nurse and other healthcare professional home visits; care from the ambulance service and nurse telephone/online appointments.

### Scoring results

Agreement between the 3 independent raters was 92% which represents almost perfect agreement. Errors (labelled issues hereafter) and struggles are presented by participant in Table 1. Most question parts (80%) were judged to have no issue or struggle. The number of issues as a percentage of question parts scored increased from Round 1 (core, 16%) to Round 2 (core or core and depth, 22%) and then decreased in Round 3 (core and depth, 9%). The most common issue was in comprehension with 28 issues across 6 participants, but the majority of comprehension issues [20] were made by one participant. The issue experienced most commonly by participants occurred while formatting the information into a valid response, with 7 participants and a total of 18 issues.

**Table 1:** Issues and struggles, by participant

Round	ModRUM version	Participant	Number of parts scored	Issues^**a**^						Struggles
				C	Ret	J	Res	U	Total	
1	Core module	1	9	–	–	–	2	1	3	1
		2	9	–	–	–	2	–	2	–
		3	9	2	–	–	–	–	2	2
		4	9	–	–	–	–	–	–	2
		5	9	–	–	–	–	–	–	–
		*Total*	*45*	*2*	*–*	*–*	*4*	*1*	*7*	*5*
2	Core module	6	9	–	4	1	–	–	5	2
		7	9	–	1	–	1	–	2	–
		8	9	1	1	–	3	–	5	1
		*Total*	*27*	*1*	*6*	*1*	*4*	*–*	*12*	*3*
	Core module and depth questions	9	31	–	–	2	–	–	2	1
		10	31	1	–	2	3	–	6	–
		11	31	–	3	–	–	–	3	–
		12	31	–	–	–	–	–	–	2
		13	31	20	–	–	–	–	20	1
		14	31	–	–	2	1	–	3	5
		*Total*	*186*	*21*	*3*	*6*	*4*	*–*	*34*	*9*
3	Core module and depth questions	15	34	–	–	–	–	–	–	2
		16	34	1	–	3	–	–	4	1
		17	34	–	–	1	–	–	1	1
		18	34	–	–	–	–	–	–	–
		19	34	–	–	–	–	–	–	–
		20	34	3	4	–	6	–	13	–
		*Total*	*204*	*4*	*4*	*4*	*6*	*–*	*18*	*4*
*All*		*Total*		*28*	*13*	*11*	*18*	*1*	*71*	*21*

^**a**^*C* comprehension, *Ret* retrieval, *J* judgement, *Res* response, *U* uncategorised issue

A summary of specific issues and struggles is provided in Additional file 1: Table S3. The most common issue/struggle experienced was uncertainty of what healthcare to include. Ambiguity on whether the questions relate to personal or dependant/family healthcare resource use also led to two comprehension issues and two struggles. The number of issues/struggles scored by healthcare item is presented in Additional file 1: Table S4. For the core module, issues were scored for the outpatient question most often. For the core module plus depth questions, issues were scored most often for prescribed medication name and number of prescriptions. Changes made to ModRUM between and during rounds are presented in Additional file 1: Table S5.

#### Comprehension

Most comprehension issues were due to participants including their dependant’s/family’s healthcare use in their response.

Participant (P)3: *For myself, it would be none. For others, I think at least once. So, would you like- I’m just going to put one.*

P13: *I’m the person responsible for them and it’s a process that I’m heavily involved with. I am going to include my interactions on my kids’ behalf.*

An attempt to clarify this issue was made following round 2, by including ‘healthcare you, yourself, have used’ in the instructions. However, in round 3, P15 was still uncertain whether to include their dependant’s healthcare, so following the interview with P16, an additional sentence was added to the instructions (‘Please do not include any healthcare your family or dependants have used’) and no further issues/struggles were scored for this reason.

There was also evidence of some comprehension issues surrounding the terminology used to describe healthcare professionals, with two participants reporting nurse consultations under GP consultations and one participant acknowledging when probed that they did not understand the term ‘outpatient’ resulting in an incorrect response.

P20 experienced issues with the table for outpatient appointments; they interpreted examples that were within table headings as part of the question. Table formatting was revised so that examples were clearly separated from table headings on a separate row.

#### Retrieval

Retrieval-related issues included being unable to recall information and including healthcare use that occurred outside the recall period. Several participants referred to additional healthcare use during the probing questions, that they did not include during the think-aloud exercise. Two participants said they felt *“a little bit rushed … a little bit under pressure”* (P11) while completing ModRUM during the think-aloud exercise.

P7: *it’s actually quite stressful, thinking, when somebody is there.*

Several participants said they would have retrieved more information had an interviewer not been present during ModRUM completion.

P17: *put a best guess in because I would have otherwise had to have got up and disturbed the questionnaire.*

The recall period was initially specified above each section as opposed to within each question; however, P6 and P8 included appointments outside the recall period. ‘In the last 3 months’ was added to the start of each question following the interview with P8 and no further problems occurred due to this issue. Some evidence of telescoping, where events outside the recall period are ‘telescoped’ in, was revealed during probing.

P6: *The last 3 months … I think it was actually before then, thinking about it.*

P15 said *“It was hard to decipher what events were in the last three month window”*. Several other participants also reported this difficulty.

Further feedback on the recall period was sought in probing questions. Most participants thought 3 months was acceptable and four participants said they could recall healthcare use from 6 months ago. Participants reported that irregular healthcare was easy to remember.

P15: *it’s irregular for me, so it stands out in my mind.*

Mixed feedback was provided on the ease of recalling regular healthcare use. P7 and P14 said seeing different healthcare professionals for different reasons makes it harder to remember regular healthcare use.

P7: *The GP is easy because that’s long term, so every 2 weeks is actually quite easy to remember.*

P14: *I think if you’re a regular visitor to a GP surgery because of ongoing health issues you lose track of how many times … actually trying to remember in a 3 month period that’s quite hard.*

P4 said *“you always get a letter”* for secondary care appointments, so they are easier to remember, whereas for appointments at the GP practice they would have to *“look in my diary because I wouldn’t be able to remember”.*

#### Judgement

Issues in judgement occurred when participants deemed relevant healthcare irrelevant, irrelevant healthcare relevant or recorded healthcare use under an unintended question.

Three issues occurred due to healthcare being judged irrelevant; this included P16 who did not include eczema creams under medications and P9 who said they had an electrocardiogram at their cardiology appointment, but recorded ‘N/A’ under ‘Tests or surgical procedures performed’. P9 said the example (removal of a skin lesion) led to them putting ‘N/A’, as the example *“seems quite severe”* and *“an ECG doesn’t affect you … It’s not invasive”*. P9 agreed a less invasive example could be included and ‘x-ray’ was added as an example following round 2.

P10 included private physiotherapy under other healthcare professionals as the question did not specify to include NHS healthcare professionals only.

P10: *‘How many times have you had contact with any other healthcare professionals?’ Now, they’re healthcare professionals. She’s a physiotherapist, a proper physiotherapist, that’s why I filled it in*

While the instructions at the beginning of ModRUM specified that respondents should ‘include healthcare you have used as an NHS patient’, this response highlighted that further clarification, to include only include NHS healthcare, was required for questions on other healthcare professionals.

P14 recorded ‘advanced nurse practitioner’ under GP as opposed to where it was intended to be recorded under nurse. They were unsure of where to include it, but judged advanced nurse practitioners more similar to GPs, with respect to the type of consultations they provide.

P14: *I would class them more as a type of consultation I’d have with the GP.*

#### Response

Missing answers were the most common response issue and were due to the participant not seeing the question, due to the answer being zero or recalled verbally by the participant but not included in the questionnaire and without evidence of an intentional judgement to omit the information. The number of missed responses due to the answer being zero diminished to zero by round 3. Following round 1, an instruction to ‘answer all the questions, even if your answer is zero’ was inserted in bold font and an instruction was added at the end, asking participants to check that they have answered every question. Missing responses due to not seeing the question, appeared to be a feature of the stapled questionnaire. P20 said the questionnaire *“would be lot better if it was a booklet, when they turn over to the next page, then they know, it’s, ‘Oh, I’ve got two sides to fill in’”*.

#### Struggle

Struggles occurred when participants hesitated or displayed uncertainty about answering a question. This included uncertainty around where responses should be written (e.g. whether to write the type of test performed under test or reason for outpatient visit) and what should be included within a question (e.g. does a chiropodist come under another healthcare professional?). Several participants appeared to struggle in recalling information, including P4 who provided a guess for the number of GP appointments at the practice because they “*… would have to go to my diary … because I wouldn’t be able to remember … so it has to be a guess”*. Guesses were not deemed as issues as participants were advised in the instructions to include their best guess if they were unsure of an answer.

### Qualitative results

#### Terminology

Most participants suggested that they understood the term ‘outpatient’, with several referring to their own experience as outpatients. However, many participants displayed some lack of understanding as to what could occur at an outpatient appointment. Two participants thought accident and emergency (A&E) visits were included under outpatients, P2 described them as *“one-off”* appointments and P1 said *“you don’t go in there for an operation”*. Examples of outpatient appointments were added to ModRUM following Round 1, and this appeared to aid understanding.

P15: *‘outpatient’, I’m not 100% clear on what that is. I just assumed, by the example you’d given- That sort of defined that, for me.*

In ModRUM, ‘day case care’ was accompanied by a brief definition (‘used a bed, but did not stay overnight’), which many participants said was helpful. While 3 participants said they did not know the meaning of the term, most other participants described it as a hospital visit where a surgery or procedure is performed. ‘Care’ was removed from ‘day case care’ following round 2, as two participants thought the question was referring to home care.

Further uncertainty around what to include within a question was expressed for other questions. For example, P12 said they were unsure of what other healthcare professional or healthcare service at home means but suggested that the inclusion of an example could help. Participants also highlighted areas where the same services could be delivered at different locations or double counting could occur. P13 included out-of-hours under GP practice/health centre and A&E as the out-of-hours clinic was usually run from the GP practice; however, on one occasion, due to Christmas, the service was delivered from A&E. P9 included the same visit to a walk-in centre under GP practice/health centre and A&E. To mitigate this issue, ‘walk-in centre’ was added to non-hospital-based questions; for example, for appointments with a GP the revised question asks ‘how many times have you had an appointment with a doctor (GP) at a GP surgery, health centre or walk-in centre?’

#### Questionnaire instructions and design

Participants that read the instructions thought that they were acceptable and described them as *“straightforward”* (P1) and *“easy to understand”* (P4). P3 did not read the instructions which led to some uncertainty when answering the questions. They said they *“just usually scan it and just pick out the key words of what it says sometimes”* when reading instructions in general. Bold fonts were added to key points in the instructions following round 1, as when asked if it would be helpful, P3 said *“Yes... Because I’ve got learning difficulties. That’s what helps me focus, picking out the key points of things.”*. Bolding throughout the questions was added following round 2 as other participants suggested highlighting important terms throughout.

P15: *in the context of people trying to do it quite fast, it would be easy to miss a key point, and then your data is not accurate.*

There was mixed feedback on including section headings in the core module only. P5 said it could be useful as *“it clearly defines the two [sections]”*. However, other participants thought it was unnecessary, with P1 stating *“it’s laid out like that anyhow”*. For the core module plus depth questions, P13 said the depth questionnaire needed more signposting as it was unclear when answering earlier questions what healthcare would be captured in later questions. This resulted in them including midwife under nurse as opposed to other healthcare professional. P13 said: *“If I was doing the survey to do the survey for real, I would have to go back and fix it. It would take extra time, and I would find that quite frustrating.”* Following round 2, signposts, such as ‘Questions 1 to 3 ask about emergency healthcare: A&E and ambulance’, were added to each section. In round 3, while P17 said *“I didn’t feel I needed it”*, other participants found them helpful.

Response options for questions requiring numerical responses included tick boxes for 0–4 and a larger box for more than 4, with the instruction ‘Please tick or write a number’. Most participants used the response options as intended and feedback was generally positive; however, there was some confusion about the large box. ‘Other’ was added above the large box following round 2; however, P20 said *“I wouldn’t put ‘Other’”*, so this was updated to ‘How many?’ following round 3.

One of the aims of participant testing was to identify areas where respondent burden could be minimised, while maintaining sufficient detail for precise estimation of costs. In the depth secondary care questions, tables capture detail on items including ‘clinic type’, ‘reason’ and ‘tests or surgical procedures’. Participants were asked about combining ‘reason’ and ‘tests or procedures’. As two participants said they would provide less detail and participants were generally positive about providing more information, items were not combined. However, they were reordered with ‘tests or procedures’ followed by ‘reason’ to rectify confusion about what to include under ‘tests or procedures’ when they had already included the test/procedure under ‘reason’.

During the interviews, some participants retrieved information on their healthcare use (including diaries, calendars, hospital letters and medications) and others stated they would have retrieved them if an interviewer had not been present. P16 got up twice during the think-aloud to retrieve information, and agreed during probing that some instruction at the beginning, to have the information *“handy … would be a good idea”*. The research team agreed that in a ModRUM user guide, researchers could be advised to include this in a cover letter to accompany ModRUM.

#### Acceptability

Most participants provided positive feedback on the length, content and layout of ModRUM. When probed, most participants did not report difficulty completing the questionnaire, P19 said it was *“quite self-explanatory”* and P11 said they *“didn’t find anything confusing”*. P20 had trouble as they *“don’t have a very good memory”* due to a stroke. They also found examples unhelpful as *“at my age, thirties, downwards, wouldn’t know what that means”*. All but one participant, who completed the longer version of ModRUM, thought the length was acceptable.

P7: *I was expecting it to be quite long, so that was actually quite easy.*

However, P14 and P17, who completed the core module plus depth questions version of ModRUM, indicated that it may not be acceptable had they used more resources in the past 3 months.

P14: *I’ve had two really significant surgical procedures … had the 3 months fallen either side of those things I would have been writing forever I suppose.*

## Discussion

### Statement of principal findings

ModRUM is a newly developed, generic RUM that once fully validated can be used to capture resource-use data in a wide range of RCTs in a standardised format, which will increase comparability of health economic research. In cognitive interviews with a range of patients recruited from primary care, most participants reported that the content, length, and layout of ModRUM was acceptable. Participants generally provided responses to questions consistent with what the questions were intended to measure, and issues were not scored for the majority (80%) of questions, providing evidence for the content validity of ModRUM. Issues identified were used to iteratively refine and enhance the comprehensibility and acceptability of ModRUM.

### Comparison to existing literature

ModRUM differs from existing RUMs as it is a concise, generic RUM designed to collect resource-use data in a standardised and consistent manner, with a modular design allowing flexibility to ensure it is relevant for a wide range of RCTs. While validation is rarely undertaken for self-report RUMs used in RCTs [5], the development process of ModRUM has been extensive and is ongoing. The Client Service Receipt Inventory is the most commonly used RUM [24]; however, it differs from ModRUM as it was designed for interviewer-administration to capture resources related to mental health conditions and has subsequently been adapted many times inhibiting standardisation in implementation [25].

Interviews with patients have been performed in the development of some RUMs. Patient interviews have informed item identification [26, 27] and item formulation [28], and similar to this study they have also been used to identify problems encountered during RUM completion [10, 11].

Ruof et al. (2004) performed in-depth interviews with patients to identify the appropriate level of aggregation for items included in their RUM for capturing resource use from patients with rheumatoid arthritis [28]. Each patient was shown various levels of aggregation for each item and feedback tended towards higher levels of aggregation from which a preliminary version of the RUM was developed [28].

Chernyak et al. (2012) developed a RUM for capturing resource-use data from patients with diabetes mellitus [10]. The RUM was tested in cognitive interviews with 43 patients, of which 19 tested a self-administered version and 24 tested an interviewer-administered version [10]. Like this study, the researchers undertook behaviour coding of interview transcripts to identify problems experienced by participants in answering questions and refined the RUM in response to comprehension and recall problems identified [10].

‘Think-aloud’ interviews with concurrent and retrospective verbal probing were also used in a study to refine an adapted version of the Client Service Receipt Inventory, designed for proxy-completion by bereaved relatives of cancer patients [11]. Nine interviews were conducted and revealed comprehension issues due to difficulty in deciding what to include under each group of healthcare services and retrieval issues related to uncertainty around the number of contacts, and information on hospital wards and medications [11]. Subsequent refinements included asking for less detail and providing examples [11].

### Strengths and weaknesses of the study

We were able to recruit 20 patients with a broad range of characteristics using a purposeful sampling strategy. While we attempted to recruit patients from harder to reach groups, we were more successful with some than others. We were unable to recruit any patients from non-white ethnic groups and as a result, this study does not provide evidence for the acceptability and content validity of ModRUM for patients in this group. Younger patients were also slightly underrepresented, which may have been due to recruiting patients attending GP appointments, for which younger patients tend to attend less frequently. As ModRUM was developed for use in one healthcare system, international use of ModRUM would require translation and content validation prior to implementation.

Concurrent interviews and analysis allowed for revisions to ModRUM and the topic guide to be made iteratively and in response to interview findings, with revisions tested in subsequent interviews. For difficulties that were revealed during the think-aloud exercise, follow-up questions allowed a greater understanding of these issues and in some cases, how they could be improved or rectified. Verbal probing complemented the think-aloud task as it allowed us to gain valuable feedback on the design, formatting and length of ModRUM, from which we were able to make refinements. Refinements were generally consistent with recommendations from questionnaire design literature on making behavioural questions easier to answer [29]. For example, aided recall, in the form of examples, was increased to aid comprehension, and who the questions were referring to was initially clarified by including ‘you, yourself’, then subsequently clarified by stating that respondents should not include family healthcare use. The 3-month recall period allowed identification of issues at all stages of the cognitive process of answering questions, whereas a longer period may have limited the identification of issues in the judgement and response processes as issues would have been more likely to occur in the retrieval process.

Face-to-face interviews are beneficial as they allow the interviewer and participant to build rapport prior to undertaking the research activity [30]. However, interviewer-bias was evident in this study as two participants reported feeling under pressure while completing ModRUM in front of an interviewer and while thinking-aloud. Some participants also said they would have referred to their diaries/medical notes had an interviewer not been present. The artificial aspect of completing ModRUM during a think-aloud exercise does not reflect how it would be implemented in RCTs. As a result, the true number of issues in usual administration of ModRUM is likely to be different than observed in this study. The issues and struggles that occurred due to confusion over whether to include family/dependant resource-use may not have been an issue in an RCT setting, as participants are more likely to be aware that they are required to report their own resource use only. However, in an RCT, RUMs are often nested within a large booklet of outcome measures which may mean participants become fatigued and are more likely to rush through the RUM, leading to missing or incorrect responses.

When expressing interest in taking part, patients were asked to provide details on the frequency of primary and secondary care use in the past 3 months. The aim of this was to recruit patients who were active healthcare users. Responses to ModRUM indicated that some resources were well utilised by participants, yet others including inpatient and day case stays, home visits and care from the ambulance service, were used by a minority or no participants. More issues were scored for resources that were used by more participants, such as outpatient appointments and prescribed medications. The cognitive process of answering questions was less clear when participants had not used a resource as responses were often immediate, as these responses generally did not require much thought. As issues with these questions may not have been revealed, it is difficult to draw conclusions on the validity of these questions for patients who use these resources.

### Implications for research practice

While self-reported RUMs have frequently been used as the primary source of resource-use data used within RCTs, the advent of routinely collected electronic data has provided an alternative method [25]. However, as accessing routine data can be costly and time consuming, at present self-report RUMs may be the optimal method for collecting resource-use data [25].

Evidence of item identification, validation and piloting of existing RUMs is limited [5]. As a result, researchers should consider potential measurement error when collecting and interpreting data captured via RUMs developed without thorough testing. Consideration should also be given to whether measurement error is likely to be systematic (leading to decreased precision of cost estimates in both arms) or differential (leading to biased estimates of incremental costs between treatment arms).

In line with existing literature, we found that the ability to recall resource-use data was impacted by many factors including the recall period, resource type and frequency of use [31]. A 3-month recall period was used in this study as it is a commonly used recall period in RCTs [32]. However, as economic data collection points are often determined by outcome measurement time points in RCTs, researchers will be able to adapt the ModRUM recall period as appropriate for their study. For alternative recall periods, researchers should consider implications to the acceptability for respondents and the validity of responses as the accuracy of results may diminish as the recall period increases [33].

### Unanswered questions and future research

As the content and face validity of ModRUM have been established, the remaining measurement properties can be tested [1]. A larger, quantitative patient pilot study would allow measurement properties including the feasibility, acceptability (completion and response rates), construct validity, criterion validity and reliability of ModRUM to be tested; in a setting that is more akin to how ModRUM would be administered in RCTs (i.e. without an interviewer present).

As many RCTs take a perspective beyond the healthcare sector, items for development into bolt-on modules that could add breadth to the core module were identified in the Delphi consensus survey [7]. Development of breadth modules would ensure that resources beyond healthcare are captured in a manner consistent with healthcare resources. Modules for development could include social care, residential care, informal care and personal expenses. Development of a remote access module for capturing online and telephone appointments could also be prioritised, considering increased utilisation of telecommunications technology to access care at present and potentially into the future.

As we were unable to recruit any patients from non-white ethnic groups, future qualitative research focusing on harder to reach groups would allow statements to be made about the acceptability validity of ModRUM for these groups. An alternative patient identification and recruitment strategy may be needed to reach these patients. Further research could also consider how issues may vary according to health conditions. As ModRUM is a generic RUM, patients were recruited without consideration to the conditions they may have; however, one participant revealed that their memory was impaired due to a stroke. Patient groups to focus on could include patients with impaired cognition (e.g. dementia), where development of a simplified or proxy-version may be more suitable and patients with high resource-usage (e.g. end of life care) where the RUM-completion burden may be heightened.

Many of the struggles identified related to uncertainty surrounding what to include within each question. Within a paper-based RUM the ability to provide an exhaustive list of the examples is limited without significantly increasing the length and potentially hindering the comprehensibility of the questionnaire. An online version of ModRUM could enhance comprehension by including drop down lists of examples and/or definitions provided when respondents hover over terms. An online version could also increase uptake of ModRUM in RCTs, as data collection methods move away from paper-based to online formats.

## Conclusion

A generic, well-validated RUM is needed to increase standardisation, comparability, and validity of health economic research. ModRUM is a newly developed, generic RUM. Using cognitive interviewing, the content validity and acceptability of the content, length, and layout of ModRUM have been demonstrated.

## Supplementary Information

**Additional file 1.**

## Data Availability

The datasets used and analysed during the current study are available from the corresponding author on reasonable request.

## Abbreviations

RCT: Randomised controlled trial
RUM: Resource-use measure
ModRUM: Modular resource-use measure
NHS: United Kingdom National Health Service
GP: General Practitioner
PCO: Primary care organisation
A&E: Accident and emergency

## Footnotes

1 User-defined weights were as follows: agreement = 1, different errors scored = 0.5, struggle versus no error = 0.5, error versus struggle = 0.25, error versus no error = 0
