# Supplementary material for: Development of a brief, generic, modular resource-use measure (ModRUM): cognitive interviews with patients
Source: BMC Health Serv Res. 2021 Apr 21;21:371. doi: 10.1186/s12913-021-06364-w (PMC8058988; doi:10.1186/s12913-021-06364-w)
Supplement: Supplementary file 1 — Additional file 1. [file 12913_2021_6364_MOESM1_ESM.pdf]

Participant Identification Number:

University of BRISTOL

Development of a healthcare use questionnaire

Your use of healthcare services

We would like you to answer some questions about the healthcare you have used, for any reason, in the last 3 months. We only want you to include healthcare you have used as an NHS patient.

Please answer all the questions, even if your answer is zero, as it is important for us to find out what healthcare you have and have not used. If you are unsure of an answer, please write your best guess.

PRELIMINARY DRAFT

In the last 3 months,	Please tick or write the number of times
1. How many times have you been to a hospital Accident and Emergency (A&E) department?	0 ☐ 1 ☐ 2 ☐ 3 ☐ 4 ☐ ☐
2. How many times have you received care from the ambulance service:	
and were taken to hospital?	0 ☐ 1 ☐ 2 ☐ 3 ☐ 4 ☐ ☐
but were not taken to hospital?	0 ☐ 1 ☐ 2 ☐ 3 ☐ 4 ☐ ☐

This is a preliminary facsimile of ModRUM; finalised versions will be released in due course. Please contact us to register your study before using ModRUM.

In the last 3 months, Please tick or write the number of times

3. How many times have you been to hospital for an outpatient appointment? 0 ☐ 1 ☐ 2 ☐ 3 ☐ 4 ☐ ☐

For each appointment, please complete the table below:

Outpatient appointment	Clinic type e.g. Dermatology	Reason for visit e.g. Mole removal	Tests or surgical procedures performed (if applicable) e.g. Removal of a skin lesion
1			
2			
3			
4			
5			
6			

If you need more space, please use the box on page 8.

PRELIMINARY DRAFT

This is a preliminary facsimile of ModRUM; finalised versions will be released in due course. Please contact us to register your study before using ModRUM.

In the last 3 months, Please tick or write the number of times

4. How many times have you been to hospital for day case care (used a bed, but did not stay overnight)?

0 ☐ 1 ☐ 2 ☐ 3 ☐ 4 ☐ ☐

For each visit, please complete the table below:

Hospital day case visit	Department e.g. Gastroenterology	Reason for visit e.g. Stomach ache	Tests or surgical procedures performed (if applicable) e.g. Endoscopy with biopsy
1			
2			
3			
4			
5			

If you need more space, please use the box on page 8.

This is a preliminary facsimile of ModRUM; finalised versions will be released in due course. Please contact us to register your study before using ModRUM.

PRELIMINARY DRAFT

In the last 3 months,

Please tick or write the number of times

5. How many times have you been to hospital for an overnight stay?

0 ☐ 1 ☐ 2 ☐ 3 ☐ 4 ☐ ☐

For each stay, please complete the table below:

Hospital overnight stay	Number of nights spent in hospital	Department e.g. Orthopaedics	Reason for stay e.g. knee replacement	Tests or surgical procedures performed (if applicable) e.g. Knee replacement
1	☐ nights			
2	☐ nights			
3	☐ nights			
4	☐ nights			
5	☐ nights			

If you need more space, please use the box on page 8.

This is a preliminary facsimile of ModRUM; finalised versions will be released in due course. Please contact us to register your study before using ModRUM.

PRELIMINARY DRAFT

In the last 3 months,	Please tick or write the number of times
6. How many times have you had an appointment with a doctor (GP):	
at a GP surgery or health centre?	0 ☐ 1 ☐ 2 ☐ 3 ☐ 4 ☐ ☐
at home?	0 ☐ 1 ☐ 2 ☐ 3 ☐ 4 ☐ ☐
over the telephone or online?	0 ☐ 1 ☐ 2 ☐ 3 ☐ 4 ☐ ☐
7. How many times have you had an appointment with a nurse:	
at a GP surgery or health centre?	0 ☐ 1 ☐ 2 ☐ 3 ☐ 4 ☐ ☐
at home?	0 ☐ 1 ☐ 2 ☐ 3 ☐ 4 ☐ ☐
over the telephone or online?	0 ☐ 1 ☐ 2 ☐ 3 ☐ 4 ☐ ☐

PRELIMINARY DRAFT

This is a preliminary facsimile of ModRUM; finalised versions will be released in due course. Please contact us to register your study before using ModRUM.

In the last 3 months, Please tick or write the number of times

8. How many times have you had contact with any other healthcare professionals (e.g. physiotherapist) or healthcare services (e.g. NHS 111):

	0	1	2	3	4	
at a GP surgery or health centre?	☐	☐	☐	☐	☐	☐
at home?	☐	☐	☐	☐	☐	☐
over the telephone or online?	☐	☐	☐	☐	☐	☐

For each healthcare professional you have seen and/or healthcare service you have used, please complete the table below:

Healthcare professional or healthcare service contact	Healthcare professional seen (e.g. physiotherapist) or healthcare service used (e.g. NHS 111)
1	
2	
3	
4	
5	

If you need more space, please use the box on page 8.

This is a preliminary facsimile of ModRUM; finalised versions will be released in due course. Please contact us to register your study before using ModRUM.

PRELIMINARY DRAFT

In the last 3 months, Please tick

9. Have you picked up or received any prescribed medications?

Yes ☐ No ☐

For each prescribed medication, please complete the table:

Prescribed medication	Name of prescribed medication<br>e.g. Tramadol<br>(if you don't know the name, please put the type of medication e.g. Painkiller)	Number of times the medication has been picked up and/or received
1		☐ times
2		☐ times
3		☐ times
4		☐ times
5		☐ times
6		☐ times
7		☐ times
8		☐ times
9		☐ times
10		☐ times

If you need more space, please use the box on page 8.

This is a preliminary facsimile of ModRUM; finalised versions will be released in due course. Please contact us to register your study before using ModRUM.

PRELIMINARY DRAFT

If you run out of space in the tables, please use the box below to write about the other healthcare you have used:

PRELIMINARY DRAFT

This is a preliminary facsimile of ModRUM; finalised versions will be released in due course. Please contact us to register your study before using ModRUM.
